# Towards a reduced order model of the periodontal ligament

**DOI:** 10.1038/s41598-025-88767-x

**Published:** 2025-02-17

**Authors:** Albert Heinrich Kaiser, Christoph Bourauel

**Affiliations:** 1 Institute for Numerical Simulation, University Bonn, Friedrich-Hirzebruch-Allee 7, 53115 Bonn, Germany; 2 Oral Technology, University Hospital Bonn, Welschnonnenstr. 17, D 53111 Bonn, Germany

**Keywords:** Periodontal ligament (PDL), Orthodontic tooth movement (OTM), Test and simulation, Poro-visco-hyperelastic model, Reduced order model, Dimensionless analysis, Medical research, Mathematics and computing

## Abstract

Based on previous in vitro experiments with specimens of porcine mandibular premolars, the simulation of the periodontal ligament response to force in the initial phase of orthodontic tooth movement is described. The initial response of the periodontal ligament can be simulated with a poro-visco-hyperelastic model. For the ground substance a hyperelastic constitutive model for compressible material was used. To facilitate parameter identification a reduced order model and an optimal interpolation metamodel were used. Parameters for the constitutive model identified herein are in good agreement with published values. They indicate a high initial compressibility of the periodontal ligament, which may be attributed to the compressibility of the vascular system within the periodontal ligament. Dimensionless analysis suggests that poroelastic behaviour will gradually cease when viscoelastic relaxation progresses. This was observed as well in the simulation and confirmed by varying the poroelastic model parameters within physically justified limits. Alveolar bone permeability has a significant influence on the flow of pore fluid in the periodontium due to poroelasticity. It is argued that in vivo alveolar bone perforation may adapt locally to optimise for the predominant load situation. A strain rate hardening effect was observed, which is not covered by the simulation, and may be the subject of further investigations.

## Introduction

**Fig. 1:**
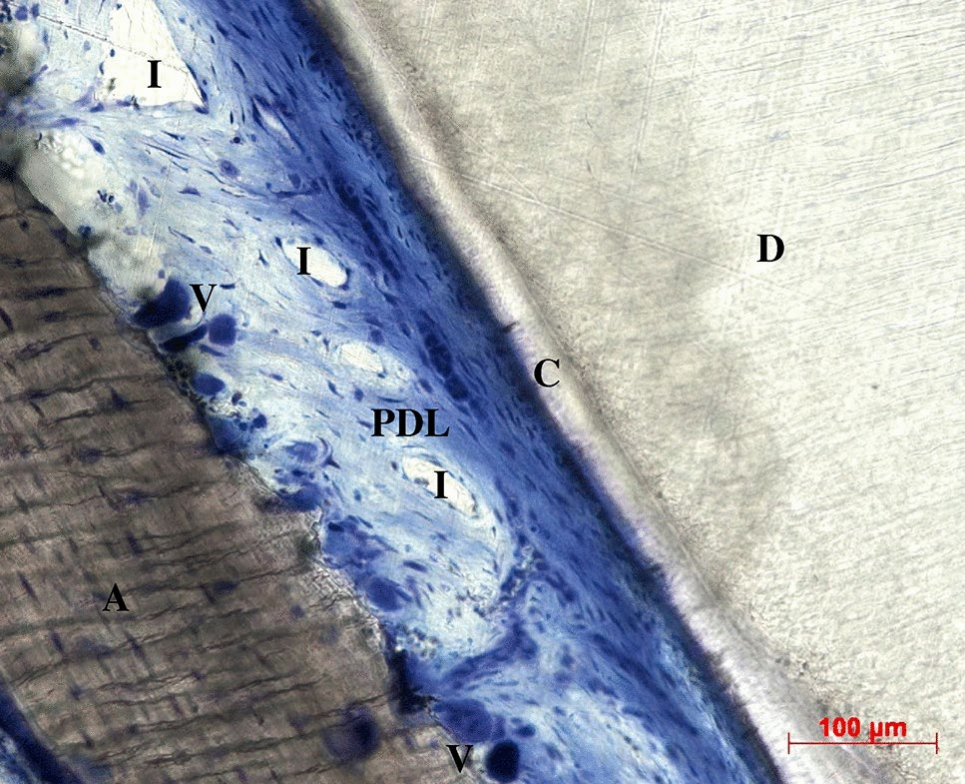
Longitudinal section of the periodontal ligament (PDL): dentin (D), cementum (C), alveolar bone (A), interstitial areas (I), blood vessels (V). Reproduced by courtesy of Werner Götz, University of Bonn.

The tissues that support the teeth, that is, the gingiva, the cementum, the periodontal ligament (PDL) and the alveolar bone proper constitute the periodontium. ‘The periodontal ligament attaches the tooth root to alveolar bone, and it serves to absorb and resist the forces of occlusion on the tooth. It consists of collagenous fiber bundles ... Interstitial areas containing loose connective tissue, blood vessels, and nerves are present between the fiber bundles in the periodontal ligament. These interstitial areas are continuous with openings through the alveolar bone (Volkmann’s canals) to the marrow spaces of the alveolar process.’^1^ Figure 1 shows a section of the PDL. A literature review points out the discrepancies in modelling approaches used and the mechanical properties published^2,3^. An improved model of the PDL for simulating the biomechanical response to orthodontic loading can contribute to a better understanding of the behaviour of the periodontium under physiological and traumatic loading, which could also benefit the planning of orthodontic tooth movements.

A ligament is a soft tissue that connects bone to bone and whose mechanical function is to guide and limit the relative movement of joints^4^. Ligaments are loaded in tension. Tests are usually carried out on specimens with the axes of the collagen fibres aligned with the direction of loading. The observed stress-strain curve exhibits a progressive behaviour and three regions are distinct: toe, heel and linear^5^. Initially, small force levels are necessary to elongate the tissue (toe region). With increased load, a progressively increased force is observed (heel region). The crimped collagen fibres gradually line up with load direction. When collagen fibres are aligned, a transition into the linear region is observed and the stress-strain curve is dominated by the straightened collagen fibres. With gradual failure of highly stretched fibre bundles, drops in the stress-strain curve and ultimate failure is observed. Under dynamic load ligaments show viscoelastic behaviour, which could be due to interaction of the collagen fibres with the ground substance proteoglycans.

The PDL distinguishes from other ligaments. The common ligament is loaded in tension, whereas under forces of occlusion, in the PDL regions of tension and compression are observed. Therefore it is anticipated that compression behaviour has a significant contribution to the overall response. With respect to that, the PDL may have similarities to cartilage. A second point is the vascular system of the PDL. The associated void space may compress readily.

At this point it may be helpful to recall the basic principle of the viscoelastic and poroelastic phenomenological model. The poroelastic model assumes a pressure gradient-induced flow of the interstitial fluid. Considering a representative elementary volume (REV) a net flow across the boundary of the REV is possible. The viscoelastic model assumes Maxwell elements. They could describe a viscous interaction of the collagen fibres and the surrounding fluid. With the viscoelastic model there is no flow of the interstitial fluid across the boundary of the REV. Both effects could occur simultaneously.

In conclusion, a simulation model of the periodontal ligament should take into account the compressive behaviour, the progressive characteristic of the stress-strain curve, and the viscous behaviour. This suggests the choice of a suitable hyperelastic constitutive material model. To account for viscous effects a viscoelastic model, a poroelastic model or a combination of these models is conceivable.

This contribution deals with the simulation of the PDL response due to force in the initial phase of orthodontic tooth movement (OTM). It is based on a in vitro experiment with specimens of porcine mandibular premolars, in which the force-displacement characteristics were determined^6^. The use of pig specimens in lieu of human specimens is justified on the assumption that both are omnivores, and the PDL structure and mechanical behaviour is similar^7^. The test is summarised in Sect. 2 and the test setup is evaluated in Sect. 3 using dimensionless analyses. Section 4 describes the finite element model used for simulation as well as the reduced order modal and the optimal interpolation metamodel used to facilitate parameter identification. Section 5 briefly outlines the approach used for parameter identification. The results of the visco-hyperelastic and poro-visco-hyperelastic simulations are presented in Sect. 6 and discussed in Sect. 7. The conclusion follows in Sect. 8.

## Test data

**Fig. 2:**
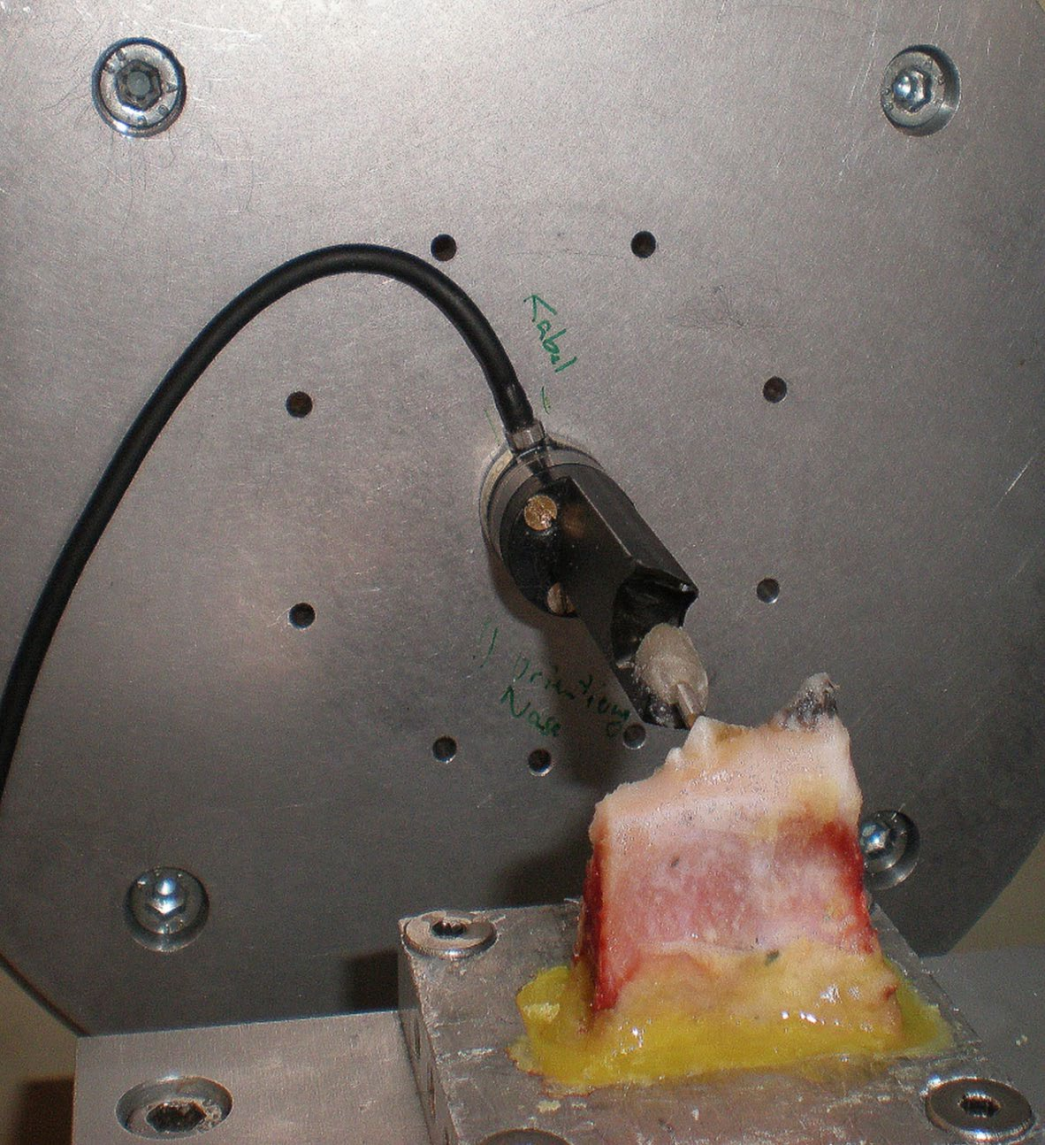
Test setup^6^: specimen mounted to the test bed with an acrylic resin, enforced displacement applied at the tooth crown (Hexapod positioning system (Hexapod M850, Physik Instrumente, Karlsruhe, Germany), a high-resolution three-dimensional (3D) force torque transducer (FTS Nano 12/120, Schunck, Lauffen/Neckar, Germany), and a 3D optical displacement measurement system (3 cameras JAI CV-M1; Stemmer Imaging, Puchheim, Germany).

**Fig. 3:**
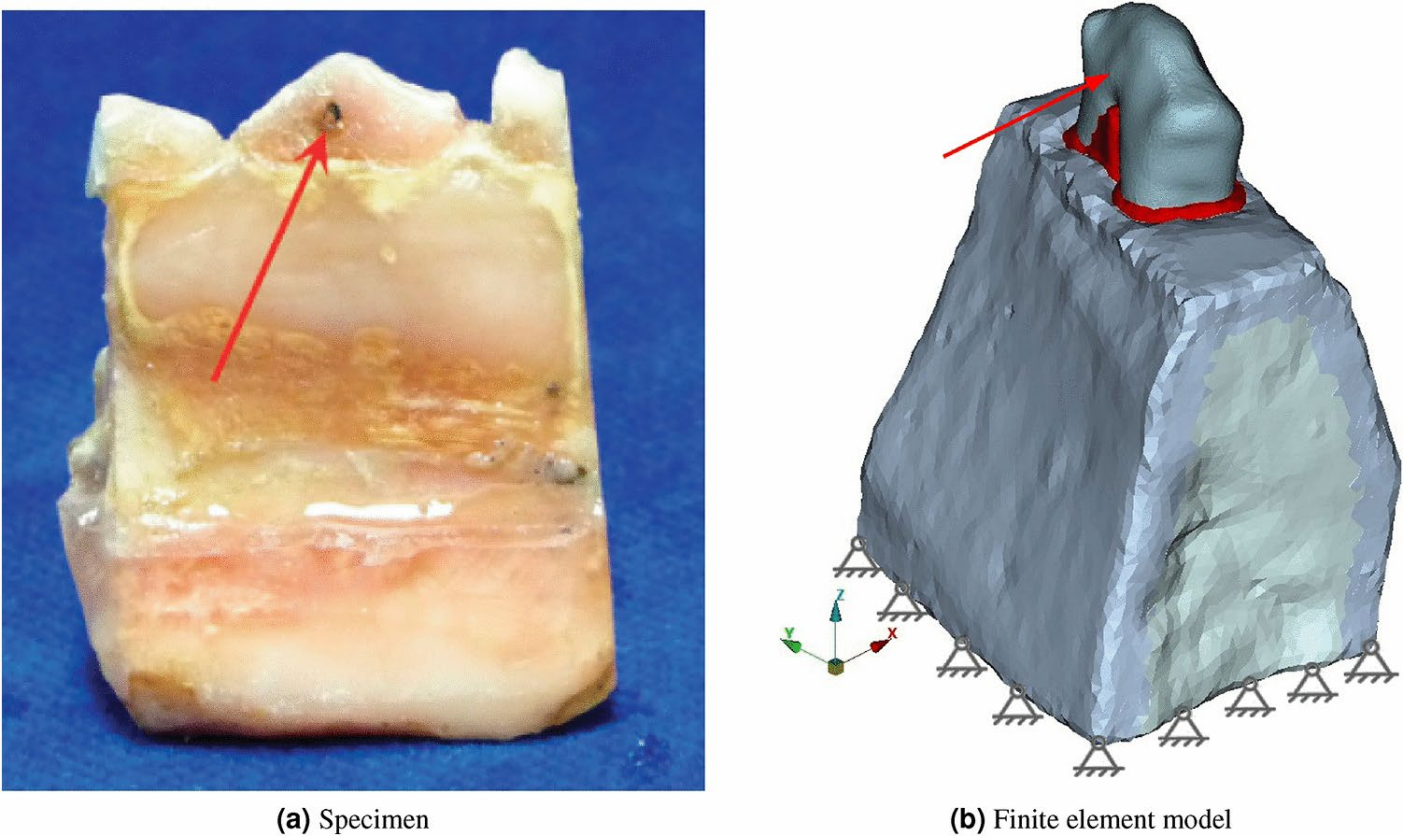
Specimen and finite element model.

The test data used are based on in vitro measurements on a specimen of a porcine mandibular premolar^6^. The specimen was fixed to the test bed with an acrylic resin (Fig. 2). Actuator displacement *x*(*t*) was input as a function of time *t* at the crown in buccal-lingual direction (Fig. 3) and the reaction force was recorded. Between $t=0$ and the ramp rise time $t=t_{ramp}$ the displacement of the actuator rose at constant speed, $v_{ramp} = d_{ramp}/t_{ramp}$, to the maximum actuator displacement $d_{ramp}$. Then, actuator displacement stayed constant until the measurement finished at time $t_{max}$ :

1 $$\begin{aligned} x = x(t)= \left\{ \begin{matrix} v_{ramp} \cdot t, & \, & 0 \le t \le t_{ramp} \\ d_{ramp}, & \, & t_{ramp} < t \le t_{max} \end{matrix} \right. . \end{aligned}$$

**Fig. 4:**
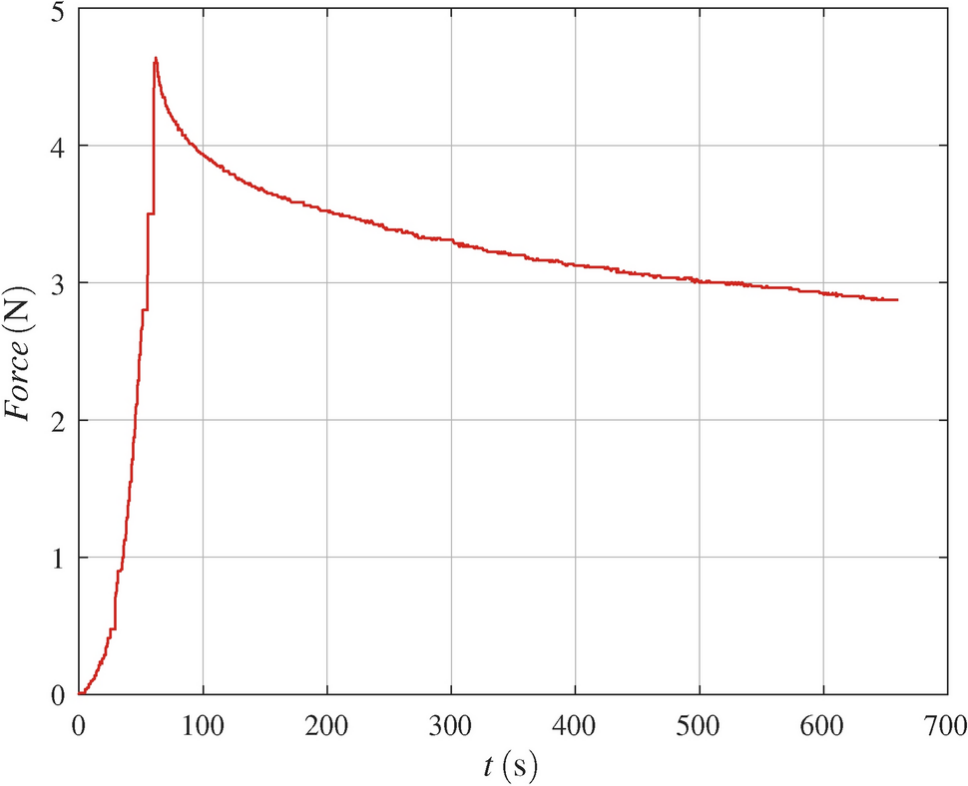
Measured actuator force versus time of test No. 10, with actuator displacement $d_{ramp} = 0.2 \hbox{mm}$ and ramp rise time $t_{ramp}={60.0}\hbox {s}$.

An overview is given in Table 1. The first column contains the test number. Then, the time of day when the test was started $t_{start}$, $t_{ramp}$, $d_{ramp}$, the sample interval $t_{sample}$, and $t_{max}$ are listed. The following two columns contain the measured peak force $F_{max}$ and the time of occurrence of this peak force $t_{Fmax}$. The last column is the rest time between the tests $t_{rest}$. As an example, test No. 10 is shown in Fig. 4. A progressive force characteristic is observed. After the maximum force is reached, a force decay with decreasing slope is observed. Due to an issue in the protocol, tests No. 1 and 13 were discarded^8^.

**Table 1:** Overview of the test data^6^.

Test	$t_{start}$	$t_{ramp}$	$d_{ramp}$	$t_{sample}$	$t_{max}$	$t_{Fmax}$	$F_{max}$	$t_{rest}$
–	hh:mm	$\hbox {s}$	$\hbox {mm}$	$\hbox {s}$	$\hbox {s}$	$\hbox {s}$	$\hbox {N}$	$\hbox {s}$
1	10:59	5	0.1	0.2	605.1	5.4	2.5	-
2	11:20	5	0.2	0.2	605.1	6.4	9.5	655
3	12:21	10	0.1	0.2	610.1	10.6	1.6	3055
4	12:36	10	0.2	0.2	610.1	10.4	8.1	290
5	12:54	20	0.1	0.2	620.1	21.0	1.0	470
6	13:14	20	0.2	0.2	620.1	20.6	6.7	580
7	13:31	30	0.1	0.2	630.1	30.6	0.9	400
8	13:49	30	0.2	0.2	630.1	30.8	5.9	450
9	14:07	60	0.1	0.2	660.2	60.5	0.7	450
10	14:30	60	0.2	0.2	660.1	61.9	4.6	720
11	14:50	120	0.1	0.5	720.0	120.2	0.5	540
12	15:12	120	0.2	0.5	720.0	123.2	4.3	600
13	15:32	300	0.1	0.5	900.3	191.3	0.4	480
14	15:57	300	0.2	0.5	900.3	305.4	3.8	600

## Dimensionless analysis

Dimensionless numbers are used to characterise physical processes. In the context of mass transfer in a continuum, the dimensionless number defined by the ratio of advection to diffusion mass flux is called *Péclet* number. The advection mass flux, i.e. the mass per time passing the boundary due to bulk fluid motion with *velocity* *v*, is $\rho _f \, v$, where $\rho _f$ is the density of the fluid. The diffusion mass flux is $D \nabla {\rho _f}$, where *D* is the *mass diffusion coefficient* (units: $\hbox {m}^{2}/\hbox {s}$). Upon replacement of the gradient $\nabla$ by a *characteristic length* *L* of the experiment, the Péclet number is

2 $$\begin{aligned} { P \hspace{-1.60004pt} e }= \frac{L \, v}{D} . \end{aligned}$$

For ${{ P \hspace{-1.60004pt} e }\gg 1}$ advection mass flux is dominant and for ${{ P \hspace{-1.60004pt} e }\ll 1}$ diffusion mass flux is dominant.

In the context of poroelasticity, advection mass flux is due to bulk fluid motion forced by a pressure gradient, usually described by *Darcy’s Law*. The diffusion mass flux is due to a mass density gradient of the interstitial fluid and described by *Fick’s Law*. For a viscoelastic material, an important characteristic of the relaxation behaviour is the time constant $\tau _v$. The test time *t* is made dimensionless by dividing through the time constant $\tau _v$, i.e. the *normalised time* is $t/\tau _v$. If in Eq. 2 velocity is replaced by $v=L/t$, the product of Péclet number and normalised time becomes

3 $$\begin{aligned} { P \hspace{-1.60004pt} e }\cdot \frac{t}{\tau _v} = \frac{L^2}{D \, \tau _v} = \frac{L^2}{L_d^2} . \end{aligned}$$

This is again a dimensionless number, defined as the quotient of the characteristic length *L* and the material-specific diffusion length $L_d$, squared. For a given test ${L^2}/{D \, \tau _v}$ is constant, and characterises the *dimensionless length* $(L/L_d)^2$ of the experimental setup. The material-specific *diffusion length*

4 $$\begin{aligned} L_d=\sqrt{D \, \tau _v} \end{aligned}$$

can be interpreted as the distance of mass transfer due to diffusion, within the time characteristic for viscoelastic relaxation.

For a given characteristic length *L* of the experiment, the diffusion time is $t_d \propto L^2/D$ and the advection time is $t_a \propto L/v$. With that, the Péclet number

5 $$\begin{aligned} { P \hspace{-1.60004pt} e }= \frac{L \, v}{D} = \frac{L^2}{D} \frac{v}{L} = \frac{t_d}{t_a} \end{aligned}$$

can be interpreted as the ratio of diffusion time and advection time, which can be considered as the *dimensionless time* of the experimental setup. If $t_d \gg t_a$ advection mass flux is dominant, and if $t_d \ll t_a$ diffusion mass flux is dominant.

Consider an in situ test of the periodontium. In the presence of an external load on the tooth, part of the interstitial fluid is likely to move to the alveolar bone. Therefore, an obvious choice for the characteristic length is the width of the periodontal ligament, which is in the order of magnitude of 0.1$\hbox {mm}$. In clinical radiology the apparent (mass) diffusion coefficient is used. The value estimated in extracranial soft tissue^9^, based on diffusion-weighted magnetic resonance imaging, is in the order of magnitude of $\hbox {10}^{-9}\hbox {m}^{2}\hbox {s}^{-1}$. The time constant $\tau _v$ is in the order of magnitude of 10$\hbox {s}$^10^. With these figures, we can estimate the constant ${L^2}/{D \tau _v}$ and find from Eq. (3) the Péclet number as a function of normalised time:

6 $$\begin{aligned} { P \hspace{-1.60004pt} e }= \frac{L^2}{D \tau _v} \, \Big ( \frac{t}{\tau _v} \Big ) ^{-1} . \end{aligned}$$

**Fig. 5:**
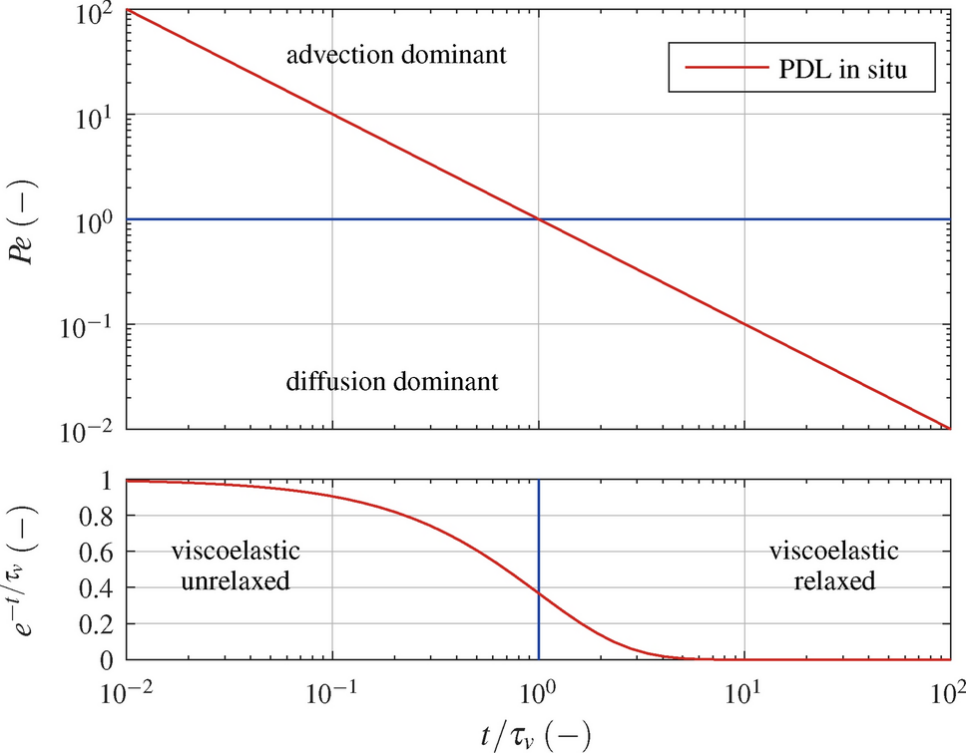
Dimensionless analysis: Péclet number ${ P \hspace{-1.60004pt} e }$ (Eq. 6) and exponential decay function $e^{- {t}/{\tau _v}}$ versus normalised time ${t}/{\tau _v}$ for the PDL in situ with $L={0.1}$mm , $D=\hbox {10}^{-9}\hbox {m}^{2}\hbox {s}^{-1}$ and $\tau _v={10}\hbox {s}$.

Figure 5 shows a plot of Eq. (6). For $t/\tau _v \ll 10^{-2}$ viscoelastic relaxation is marginal and advection, i.e. poroelastic behaviour, is dominant. For $t/\tau _v > 10^{-1}$ viscoelastic relaxation is noticeable, at $t/\tau _v = 1$ viscoelastic relaxation is in progress and at $t/\tau _v = 10$ viscoelastic relaxation is practically completed. Between $t/\tau _v =10^{-1}$ and $t/\tau _v = 10^1$ transition from advection to diffusion is observed and for $t/\tau _v > 10^1$ diffusion mass flux becomes dominant.

In conclusion, for an relaxation test of the periodontium, it is anticipated that poroelastic behaviour will be observed during the initial phase. Poroelastic behaviour will gradually cease between $t/\tau _v =0.1$ and 10. Several time constants for viscoelastic relaxation were reported^10^, and after the initial phase, viscoelastic behaviour will be observed throughout the test. It is recognised that for long times in vivo biochemical processes (orthodontic tooth movement phase two and three) and in vitro decomposition may be of significance.

## Simulation model

### Finite element model

#### Triangulation, boundary and initial conditions

A finite element model of the specimen was generated^8^. The model distinguishes tooth, PDL and alveolar, cortical and cancellous bone (Fig. 3). Plaese delete line brake here. At least two rows of elements were created across the thickness of the PDL. Element type C3D10MP^11^, i.e. 10-node tetrahedrons with displacement and pore pressure (only for poroelasticity) degree of freedom active at all nodes, modified second order interpolation of displacement and pore pressure and with hourglass control was used. During experimentation the lower third of the specimen was embedded in an acrylic resin and fixed to the test bed. At the corresponding surfaces, this was modelled with Dirichlet boundary conditions (fixed translational degree of freedom) for displacements. For poroelasticity a Neumann boundary condition (free draining) was specified at the free PDL surface, adjacent to the alveolar crest, where gingiva was removed from the specimen. At a reference point, in the centre of the actuator contact surface in the experiment, actuator displacement was applied in accordance with the test protocol (Eq. 1). The material constants used for simulation are listed in Table 2. The constitutive model of the periodontal ligament is described in the next chapter. For poroelastic simulation the initial value of pore pressure was set to zero.

**Table 2:** Summary of poroelastic constants used for mandible and tooth.

Property		Units	Cortical	Cancellous	Alveolar	Tooth
			Bone	Bone	Bone	
Young’s modulus	*E*	$\hbox {GPa}$	14.58	0.41	0.345	15.0
Poisson’s ratio	$\nu$	-	0.325	0.3	0.31	0.31
intrinsic permeability	*k*	$\hbox {m}^{2}$	$1.47 \cdot 10^{-20}$	$1.0 \cdot 10^{-9}$	$5.29 \cdot 10^{-14}$	$3.88 \cdot 10^{-17}$
porosity	*n*	-	0.05	0.8	0.8	0.05

#### Constitutive model used for the periodontal ligament

To capture the progressive characteristic of the stress-strain curve of the PDL finite-strain viscoelasticity and the hyperelastic constitutive model for compressible material proposed by Storåkers^12^ is used for the visco-hyperelastic and poro-visco-hyperelastic simulation. The corresponding strain energy density function in ABAQUS^11^ is

7 $$\begin{aligned} U\left( \hat{\lambda },J_{el} \right) = \sum _{i=1}^N \frac{2\mu _i}{\alpha _i^2} \left[ \left( \hat{\lambda }_1^{\alpha _i} + \hat{\lambda }_2^{\alpha _i} + \hat{\lambda }_3^{\alpha _i} -3 \right) + \frac{1}{\beta _i} \left( J_{el}^{- \alpha _i \beta _i} - 1 \right) \right] , \end{aligned}$$

where *N* is the number of terms used and $\mu _i$, $\alpha _i$ and $\beta _i$ are material parameters. The Jacobian *J* is a measure of the total volume change and is split into a thermal part $J_{th}$ and an elastic part $J_{el}=J/J_{th}$. The thermal volume change follows from the thermal strain, $\epsilon _{th}$, with $J_{th}=(1+\epsilon _{th})^3$. The principal elastic stretches ${\hat{\lambda }}_{k}=J_{th}^{-{{1}/{3}}}\lambda _{k}, \, k \in \{1,2,3\}$ are based on the principal stretches $\lambda _k$. The initial shear modulus is $\mu _0 = \sum _{i=1}^N \mu _i$. The coefficient $\beta _i$ determines the degree of compressibility and is related to the Poisson’s ratio, $\nu _i$, by the expressions $\beta _i = {\nu _i}/{(1 - 2 \nu _i)}$. Provided $\beta _i$ is the same for all terms, a single effective Poisson’s ratio $\nu$ is evident. The initial bulk modulus is $K_0 = \sum _{i=1}^N 2 \mu _i \left( \tfrac{1}{3} + \beta _i \right)$. Storåkers constitutive model is called in ABAQUS with the keyword *HYPERFOAM. Here, we used the strain energy density function with one term, $N=1$, and have three unknown material parameters $\mu$, $\alpha$ and $\beta$.

For the viscoelastic part, an indication of the number of terms necessary in the generalised Maxwell model, is the number of logarithmic decades spanned by the test data. This can be estimated by $n=lg(t_{max}/t_{min})$, given the maximum, $t_{max}$, and minimum, $t_{min}$, time in the test data. With the figures from Table 1 we estimate $n=lg(900/0.5)=3.26$, and choose three terms to represent the relaxation moduli in terms of the Prony series.

#### Strain-dependent permeability of the PDL

For the poro-visco-hyperelastic simulation strain-dependent permeability of the PDL was taken into account. The relation proposed^13^ and adopted in an former investigation^14^ was assumed

8 $$\begin{aligned} k=k_0 \left( \frac{n}{n_0} \right) ^2 e^{M(\lambda -1)} , \end{aligned}$$

with permeability *k*, porosity *n*, a constant $M \in \mathbb {R}^{\text {+}} \setminus 0$, and stretch $\lambda$. The subscript 0 refers to the reference state $\lambda =1$. The equation was derived for compression strains. Some researchers use it as well in tension. The authors found no evidence in the literature to support the validity of this approach. Here it is assumed that in tension permeability remains constant ($k=k_0$ for $\lambda \ge 1.0$). Measured porosity of $n = {0.70}$, an intrinsic permeability at reference configuration of $k_0 = 8.81 \cdot {10}^{-15}{m}^{2}$ and exponent $M={14.2}$ were reported^14^. These values were used here and Eq. (8) was implemented as a look-up table when specifying the permeability.

### Reduced order model

A priori the parameters of the poro-visco-hyperelastic model of the periodontal ligament are unknown. One may attempt to find optimal parameters, in the sense that the difference between measured and simulated response is minimal, by simulation with the finite element model. Due to the high number of degrees of freedom and the nonlinearities involved, this approach is computationally intensive. To address the issue, *model order reduction* methods matured in the last decades^15^. One approach, which uses physical insight to reduce model complexity, is called operational model order reduction. This path is followed here, reducing the model to one degree of freedom, corresponding to actuator displacement input.

A canonical method for solving the finite viscoelasticity problem is separation of variables^16^. By analogy, actuator force *Y*(*x*, *t*, *p*) is assumed to be the product of a function of actuator displacement $F(x,p_F)$ and a function of time $G(t,p_G)$, with time $t \in [0, t_{max}]$, and parameters $p=[p_F,p_G]$ of *F* and *G*:

9 $$\begin{aligned} Y(t,p) = F(x(t),p_F) \cdot G(t,p_G) . \end{aligned}$$

The displacement of the actuator *x*(*t*) is known from the measurement record. Consider Fig. 4, a plot of test No. 10. Right of the peak force point the displacement of the actuator is constant and a decay of the force is observed. The force appears to converge against an equilibrium value. This suggests defining $G(t,p_G)$ by analogy with viscoelastic stress relaxation models available in finite element software. As in Sect. 4.1, this leads to three exponential decay functions

10 $$\begin{aligned} G(t,p_{G}) = p_{1}+p_{2}e^{-p_{3} \cdot t}+p_{4}e^{-p_{5} \cdot t}+p_{6}e^{-p_{7}\cdot t} \,. \end{aligned}$$

The bounds of the parameters $p_G$ are $p_{i} \in (0,1)$ for $i= 1,2,4,6$ and $p_{i} \in \mathbb {R}_+ \setminus 0$ for $i = 3,5,7$. The time constants are $\tau _j=1/p_i; \, j,i \in \{(1,3),(2,5),(3,7) \}$. Assuming the smallest time constant is $\tau _1$, and that the time constants are separated by at least a factor *C*, then, the smallest time constant $\tau _{1} = 1/p_3$ should be larger than the minimum time of the measured data, i.e. the sampling interval $t_{min}$, and the largest time constant $\tau _{3} = 1/p_7$ should be smaller than the measurement time $t_{max}$. These conditions represent inequality constraints:

11 $$\begin{aligned} p_7 \ge 1/t_{max} \qquad p_3 \le 1/t_{min} \qquad \text {and} \qquad \qquad \end{aligned}$$

12 $$\begin{aligned} \frac{p_3}{p_5}&\ge C \qquad  \frac{p_5}{p_7} \ge C \qquad \qquad \text {with} \quad C=8 \,. \end{aligned}$$

As in the viscoelastic material model implemented in ABAQUS^11^, the sum of the coefficients for the viscoelastic part was chosen to be one. This is an equality constraint:

13 $$\begin{aligned} p_1 + p_2 + p_4 + p_6 = 1 . \end{aligned}$$

The response to the total load history is the sum of the individual responses due to $i=1,...,m$ displacement increments $\delta x_i$ at time $t_i$. With the unit step (Heaviside) function *u*, the displacement of the actuator *x*(*t*) is approximated by

14 $$\begin{aligned} x(t) = \sum _{i=1}^m \delta x_i \, u(t-t_i) \,. \end{aligned}$$

Now consider the response left of the peak force point. Force increases with actuator displacement *x*(*t*) and a progressive characteristic is observed. In a previous work^17^, this was modelled by the function

$$\begin{aligned} F(x(t),p_{F}) = \frac{c_1}{c_2 } (e^{c_{2}\cdot x}-1) \,, \qquad \text {with} \qquad p_{F}=[c_1,c_2] \in \mathbb {R}_+ \setminus 0 , \end{aligned}$$

which is defined analogue to the equation proposed for the quasi-static one-dimensional passive response of soft tissue in integrated form^18^.

Suppose the progressive characteristic is due to the PDL behaviour and can be modelled with a suitable hyperelastic constitutive law. Then, there should be a mapping of the measured actuator force and displacement to the stress and strain response of the PDL. At zero actuator displacement the reaction force is zero and this corresponds to the zero strain and stress point. A candidate for the mapping is to assume that strain $\varepsilon$ is proportional to actuator displacement *x*, and stress $\sigma$ is proportional to the actuator force *F*,

15 $$\begin{aligned} (F,x) \mapsto (\sigma ,\varepsilon ) = (p_8 F, p_9 x) \qquad \text {with} \qquad p_8,p_9 \in \mathbb {R}_+ \setminus 0. \end{aligned}$$

It is recognised that the stress-strain response of the PDL varies with load condition, specimen geometry and the position within the PDL. Therefore the mapping is unique for the load condition and specimen geometry on hand, and an averaged, prevailing stress-strain response is assumed. For the reduced order model the stress-strain behaviour is derived from strain energy density function, Eq. 7. With one term, $N=1$, three material parameters $\mu$, $\alpha$ and $\beta$ are unknown. Then, the initial shear modulus is $G=\mu$ and effective Poisson’s ratio is $\nu = {\beta }/{(1 + 2 \beta )}$. For an isothermal process and uniaxial state of stress the stress-stretch relation is (Ref. Appendix)

16 $$\begin{aligned} \sigma = \frac{2\mu }{\alpha } \left( 1 - \lambda ^{-\alpha \frac{1+3\beta }{1+2\beta }} \right) \lambda ^{\alpha -1}. \end{aligned}$$

With stretch $\lambda = 1 + \varepsilon$ and Eq. (15), the force, actuator displacement relation becomes

17 $$\begin{aligned} F(x) = \frac{2\mu }{p_8 \alpha } \left( 1 - (1 + p_9 x)^{-\alpha \frac{1+3\beta }{1+2\beta }} \right) (1 + p_9 x)^{\alpha -1}. \end{aligned}$$

In the in vitro test^6^ the tooth was loaded at the crown in the buccal-lingual direction. The predominant stress-strain response is then in tension and compression. That is, the reaction force caused by the actuator displacement is affected by tension and compression strains. Therefore, actuator displacement *x*(*t*) is mapped to a tension and compression stretch, $\lambda (t) = 1 \pm p_9 x(t)$, and the average of the tension and compression force contribution is used

18 $$\begin{aligned} F(x,p_F) = \frac{1}{2} ( \, F(+x) + |F(-x)| \, ). \end{aligned}$$

By virtue of this choice, estimated parameters take into account the tension and compression behaviour of the PDL. The parameters are $p_F=[p_8, p_9, \mu , \alpha , \nu ]$, with $\beta = {\nu }/{(1 - 2 \nu )}$. Collecting terms, the proposed reduced order model is:

19 $$\begin{aligned} \begin{aligned} Y(t,p)&= F(x(t),p_F) \cdot G(t,p_G) \,, \\ G(t,p_{G})&= p_{1}+p_{2}e^{-p_{3} \cdot t}+p_{4}e^{-p_{5} \cdot t}+p_{6}e^{-p_{7}\cdot t} \,, \\ F(x(t),p_{F})&= \frac{1}{2} ( \, F(+x) + |F(-x)| \, )\,, \, \text {with} \, F(x), \, \text {Eq.} \, 17. \end{aligned} \end{aligned}$$

For curve fitting of individual tests Eq. (19) is sufficient. However, when curve fitting several tests together, additional effects were observed and the reduced order model was extended by two terms. An dependency on actuator ramp rise time $t_{ramp}$ and actuator displacement *x* was taken into account with the term $H(x,p_H)$, which has two parameters $p_H=\{p_{10},p_{11}\}$. Effects of residual strains of previous tests on the current test were taken into account with the term $K(t,p_K)$. Due to the inequality constraints, the contribution of the exponential function in $G(t,p_G)$ with the smaller time constant is negligible (${e}^{-8}=3.4\cdot {10}^{-4}$), and only the contribution with the largest time constant is taken into account. The contribution of all previous tests is added up, and the deflection of the actuator is approximated by a pulse of height $d_{ramp}$ between the end of the ramp and the end of the test. For each test, time *t* starts at zero. We choose the start of test No. 1. as a fixed reference time, and provide the start time of the current test $t_{start}$, the pulse start times $t_{p s}$ and pulse end times $t_{p e}$ of the previous tests, relative to this reference. At the start of the test the measured force was set to zero, and we have to subtract $K(t=0,p_K)$ to satisfy $Y(x,t=0,p)=0$. The augmented reduced order model is then

20 $$\begin{aligned} \begin{aligned} Y(t,p)&= F(x(t),p_F) \cdot G(t,p_G) + H(x(t),p_H) + K(t,p_K) - K(t=0,p_K) , \\ G(t,p_{G})&= p_{1}+p_{2}e^{-p_{3} \cdot t}+p_{4}e^{-p_{5} \cdot t}+p_{6}e^{-p_{7}\cdot t} , \\ F(x(t),p_{F})&= \frac{1}{2} ( \, F(+x) + |F(-x)| \, ) , \, \quad \text {with} \,\: F(x),\: \text {Eq.} \, 17, \\ H(x,p_H)&= p_{10} \cdot x \cdot e^{-p_{11} \cdot t_{ramp}} , \\ K(t,p_K)&= \sum _{k=1}^{test No. -1} [ \, F(d_{ramp},p_F) \, p_6 \, ( e^{-p_7 (t_{start}-t_{ps}+t)} - e^{-p_7 (t_{start}-t_{pe}+t)} ) \, ]_k . \end{aligned} \end{aligned}$$

The parameters of $p_K = \{p_6, p_{7}, t_{start}, \{ t_{ps},t_{pe}, d_{ramp} \}_k \}$ recur from $p_G$ and are given in the test protocol. The new parameters are $p_H=\{p_{10},p_{11}\} \in \mathbb {R}_+ \setminus 0$.

In summary, a reduced order model with one degree of freedom (Eqs. 19 or 20) was derived for simulation of the reaction force as a function of actuator displacement. All parameters of the visco-hyperelastic finite element model of the periodontal ligament are incorporated in the reduced order model. Note that the scheme is discrete in time (Eq. 14).

### Optimal interpolation metamodel

Provided that for some parameter variations the finite element model was simulated and the corresponding actuator displacement and reaction force versus time data are available. Assuming the response is continuous, then there is a mapping from the model parameters to the response. For intermediate values of the model parameters the response may be estimated by interpolation. For a thorough discussion of the concept refer to textbooks^15^. In general, the (measured) data may have random content, and depending on the realm, the methods have various names, e.g. Kriging or Gaussian process model.

Here, for each of the *N* simulations with the finite element model, the *p* model parameters and corresponding reaction forces at time $t_i, \, i=1,..., m$ is known. That is, the mapping from the model parameters to the response, $\mathbb {R}^p \mapsto \mathbb {R}^m$, has *N* known instances. Intermediate values are estimated by means of optimal interpolation^19^. For this purpose, the function *optiminterp* implemented in the GNU Octave^20^ was used. On that account, the notion *optimal interpolation metamodel* is preferred.

Incidentally, in the field of artificial intelligence (AI), the parameters would be referred to as features and the response as labels. This leads directly to the use of neural networks, which requires a training phase. The approach is used in practice^21–24^. Compared to the method used here, this is computationally intensive, even more if updated simulation results are to be taken into account in an iterative process.

## Identification of optimal parameters

We want to find the parameters of the simulation model that best approximates measurement points and fulfil the equality and inequality constraints. This can be stated in terms of constrained optimisation. It is evident that evaluation of the reduced order model and the optimal interpolation metamodel are computationally cheap. Hence, optimisation was done first with the reduced order model. The thus found parameters were simulated with the the finite element model, then refined with the optimal interpolation metamodel, and successively verified by simulation with the the finite element model. Details were the subject of earlier publications^8,17^.

## Simulation results

### Individual tests with visco-hyperelastic model

Curve fits of the reduced order model Eq. (19) to individual tests were performed. Parameters of the constitutive law reported in a previous investigation^17^ were not varied. Results are listed in Table 3.

**Table 3:** Visco-hyperelastic curve fit (reduced order model Eq. 19) of individual tests with actuator displacement 0.1 mm (odd test No.) and 0.2 mm (even test No.), with parameters of the constitutive model $\mu =0.0338$, $\alpha =29.7$ and $\nu =0.236$. Final parameters, $R^2$, mean and standard deviation (S.D.).

Test	$p_1$	$p_2$	$\tau _1$	$p_4$	$\tau _2$	$p_6$	$\tau _3$	$p_8$	$p_9$	$R^2$
–	–	–	$\hbox {s}$	–	$\hbox {s}$	–	$\hbox {s}$	–	–	–
3	0.245	0.315	2.73	0.139	28.8	0.301	383.	0.0158	1.177	0.994
5	0.324	0.419	6.51	0.102	52.1	0.155	832.	0.0101	0.853	0.997
7	0.371	0.259	1.41	0.286	11.3	0.084	163.	0.0187	1.108	0.994
9	0.376	0.211	2.17	0.317	32.0	0.096	830.	0.0189	0.937	0.994
11	0.304	0.304	0.46	0.192	10.2	0.199	854.	0.0189	0.937	0.994
2	0.354	0.194	3.27	0.129	33.3	0.323	371.	0.0020	0.516	0.988
4	0.419	0.152	5.88	0.113	47.0	0.316	419.	0.0066	0.715	0.999
6	0.424	0.191	3.62	0.116	29.4	0.269	360.	0.0058	0.665	0.999
8	0.366	0.328	2.02	0.108	32.0	0.197	417.	0.0092	0.779	0.996
10	0.468	0.151	2.87	0.145	27.4	0.237	342.	0.0100	0.717	0.997
12	0.352	0.207	12.3	0.145	98.1	0.295	1171.	0.0117	0.733	0.999
14	0.338	0.366	6.86	0.102	70.2	0.194	562.	0.0101	0.749	0.997
mean	0.362	0.258	4.17	0.158	39.3	0.222	559.	0.0115	0.824	-
S.D.	0.059	0.087	3.25	0.072	24.8	0.082	296.	0.0055	0.188	-

### All tests at once with visco-hyperelastic model

**Fig. 6:**
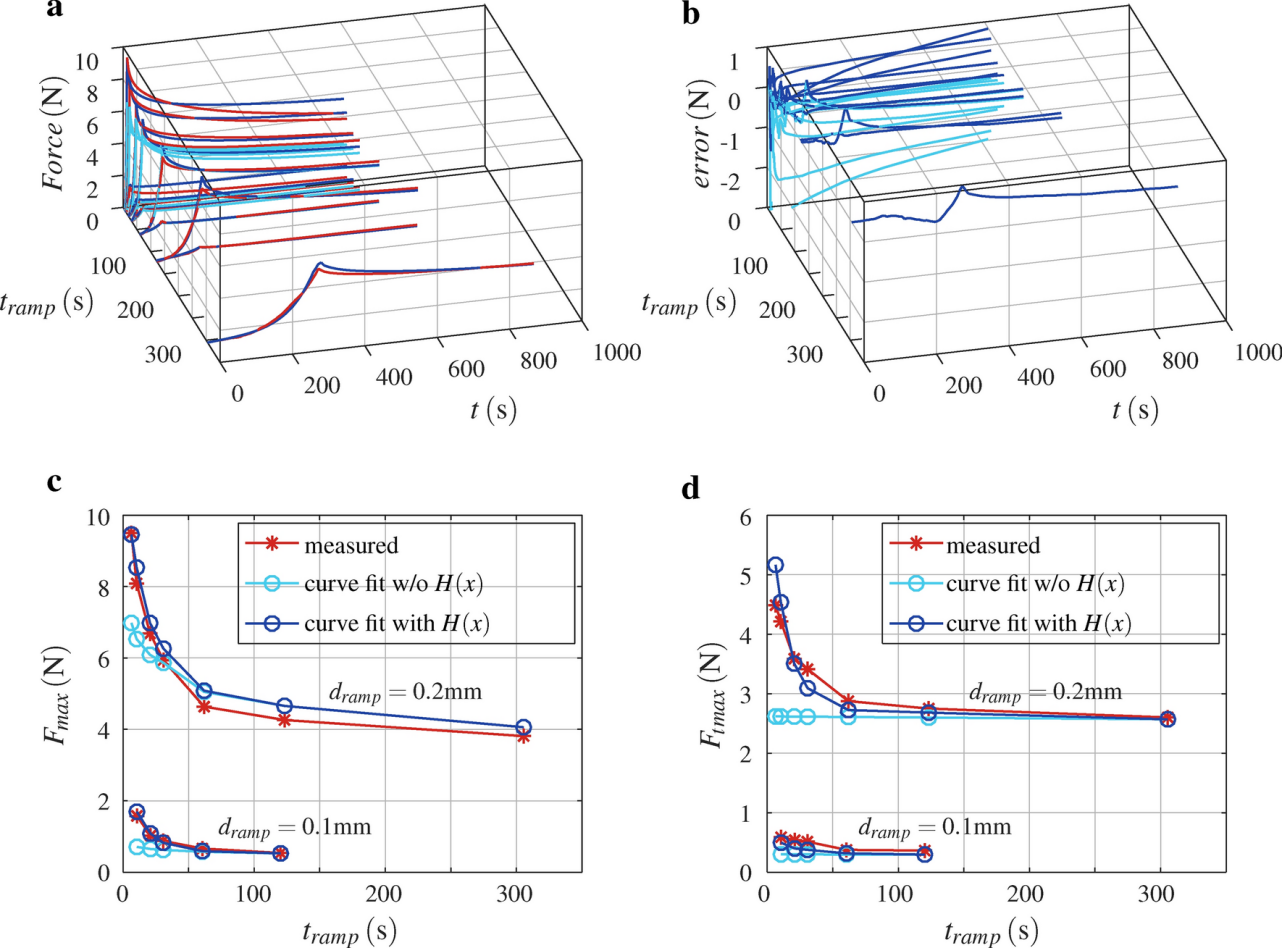
Curve fit of reduced order model Eq. (20) for the tests (Table 1, w/o No. 1 and No. 13), without $H(x,p_H)$ term $R^2=0.842$ and with $H(x,p_H)$ term $R^2=0.988$: (**a**) measured force and curve fit forces over time *t* at ramp rise time $t_{ramp}$ of the test; (**b**) error, that is curve fit force minus measured forces over time *t* at ramp rise time $t_{ramp}$ of the test; (**c**) peak force $F_{max}$, measured and curve fits, over ramp rise time $t_{ramp}$ of the tests; (**d**) force at the end of the test $F_{tmax}$, measured and curve fits, over ramp rise time $t_{ramp}$ of the tests.

Parameters for the visco-hyperelastic simulation were identified as described in Sect. 5. The final parameters are listed in Table 4. A plot of measured data and the reduced order model, Eq. (20), with final parameters, and with and without the $H(x,p_H)$ term, for all valid tests is given in Fig. 6. The coefficient of multiple correlation $R^2$ was evaluated with respect to the measured force. For the reduced order model with $H(x,p_H)$ term correlation coefficient is $R^2=0.99$, and without $H(x,p_H)$ term correlation coefficient is $R^2=0.84$. Simulated response with the finite element model and the same parameters (noting that $p_8$ to $p_{11}$ are not needed in the finite element model) has a correlation coefficient of $R^2=0.95$ with respect to the viscoelastic force from the reduced order model without $H(x,p_H)$ term, and $R^2=0.88$ with respect to measured force.

**Table 4:** Parameters of the reduced order model Eq. (20), and the visco-hyperelastic simulation (without $p_8$ to $p_{11}$), for all tests at once.

$p_1$	$p_2$	$\tau _1$	$p_4$	$\tau _2$	$p_6$	$\tau _3$	$p_8$	$p_9$	$\mu$	$\alpha$	$\nu$	$p_{10}$	$1/p_{11}$	$R^2$
–	–	$\hbox {s}$	–	$\hbox {s}$	–	$\hbox {s}$	–	–	–	–	–	–	$\hbox {s}$	–
0.320	0.275	4.52	0.160	39.1	0.245	387.	.0182	0.889	.0338	29.7	0.236	22.9	12.6	0.988

### All tests at once with poro-visco-hyperelastic model

**Fig. 7:**
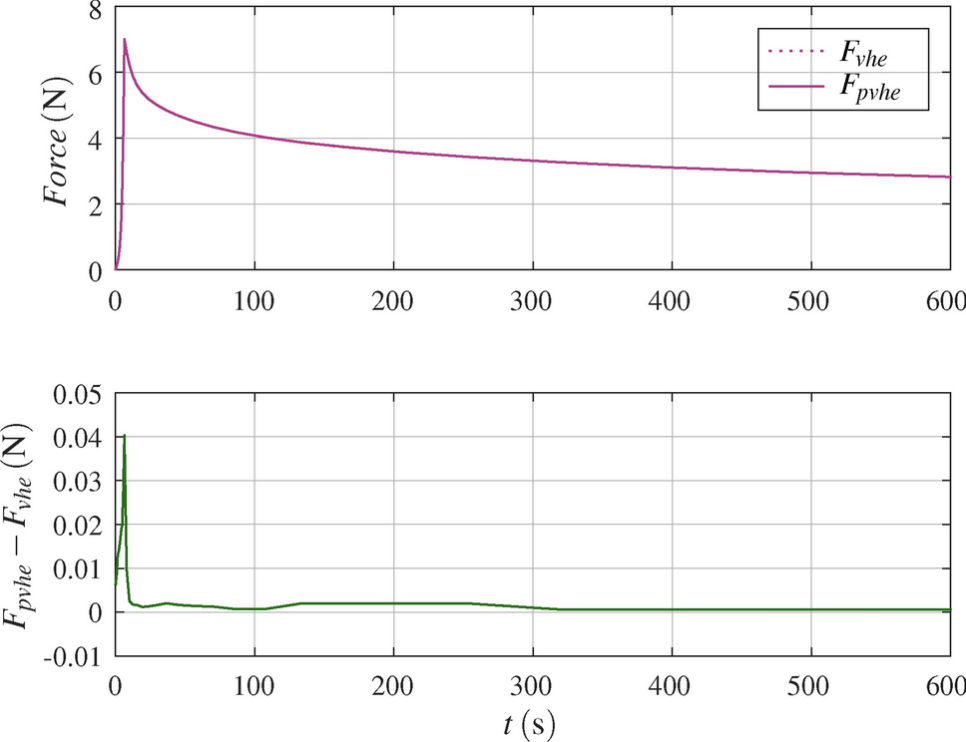
Poro-visco-hyperelastic response $F_{pvhe}$ ($k_0=8.81 \cdot {10}^{-15}{m}^{2}$, ${M={14.2}}$) versus visco-hyperelastic response $F_{vhe}$, with parameters from Table 4, for test No. 2 in Table 1, with actuator displacement $d_{ramp}={0.2}\hbox{mm}$ and ramp rise time $t_{ramp}={5.0}\hbox {s}$.

**Fig. 8:**
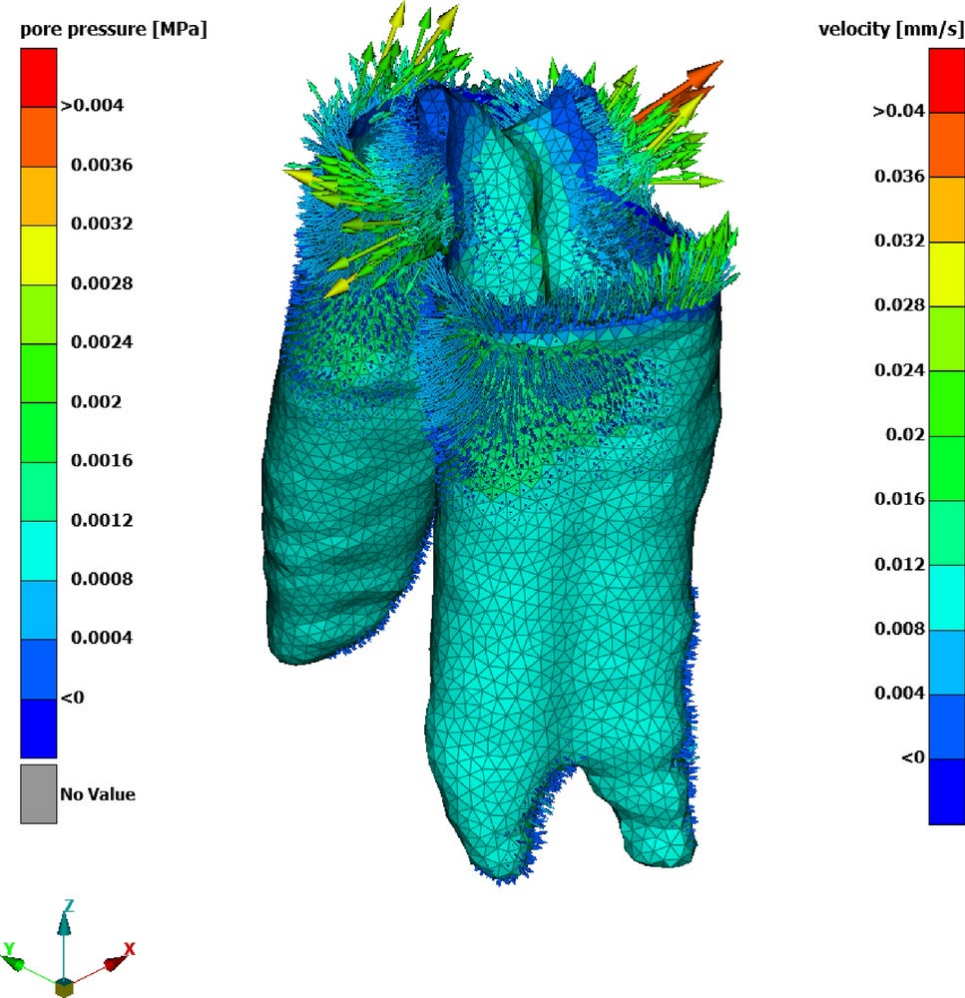
Poro-visco-hyperelastic simulation of test No. 2 in Table 1, with actuator displacement $d_{ramp}={0.2}\hbox {mm}$ and ramp rise time $t_{ramp}={5.0}\hbox{s}$. Pore pressure and pore fluid velocity at $t={5.0}\hbox {s}$, with parameters from Table 4 and $k_0=8.81 \cdot {10}^{-15}{m}^{2}$, $M={14.2}$.

**Fig. 9:**
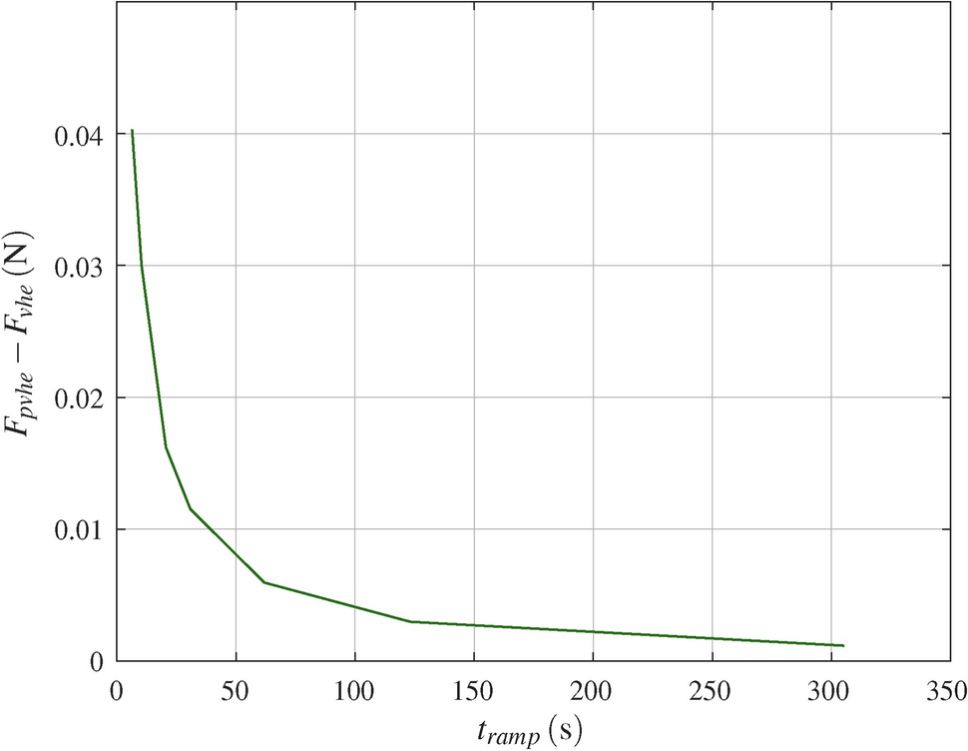
Difference between poro-visco-hyperelastic $F_{pvhe}$ and visco-hyperelastic $F_{vhe}$ peak force versus ramp rise time $t_{ramp}$, for all tests with actuator displacement 0.2$\hbox {mm}$.

The poro-visco-hyperelastic simulation is based on the visco-hyperelastic model with final parameters. In addition, strain-dependent permeability and the Neumann boundary condition (free draining), as described in Sect. 4.1, was used. A plot of the poro-visco-hyperelastic response versus the visco-hyperelastic response for test No. 2 is shown in Fig. 7. For the poro-visco-hyperelastic simulation pore pressure and velocity at $t=t_{ramp}$, i.e. at peak force $F_{max}$, is shown in Fig. 8 and an animation is found in the supplementary material. The difference between poro-visco-hyperelastic and visco-hyperelastic peak force versus ramp rise time for all tests with actuator displacement 0.2$\hbox {mm}$ is shown in Fig. 9. With the chosen parameters, the maximal difference at peak force is about 0.04$\hbox {N}$.

## Discussion

Simulation of the finite element model is computationally intensive. That is why a reduced order model and a optimum interpolation metamodel was used to facilitate parameters identification. The conception of the reduced order model supported thinking about the physical effects involved. The reduced order model allowed a full factorial experiment^25^ (analysis) at low cost. The admissible parameter range was scanned. If the coefficient of multiple correlation $R^2$ value was larger than a threshold, a Levenberg-Marquardt curve fit was executed. The results were evaluated for the region with highest $R^2$ value and there the response was simulated with the finite element model. With the result of the finite element model a refined parameter study was conducted by means of optimal interpolation. Optimal interpolation is computationally cheap. An additional advantage over neural networks is that in an iterative process new simulation results can be incorporated directly, without repeating the training phase.

Referring to Table 3, measured force, actuator displacement response are approximated well with the reduced order (visco-hyperelastic) model Eq. (19) and parameters identified for individual tests. For all tests, the correlation coefficient is larger than $R^2=0.99$. Note that identified parameters vary considerably between individual tests and standard deviations are large. Similar observations were made by other researchers^10^, who fitted a function to individual tests. Here, all tests were made with the same specimen, and for tests with the same actuator displacement one might expect a lower variation in estimated parameters. Given the parameter variation between individual tests, no merit was anticipated to further refine the parameters by simulation with the finite element model.

The final parameters identified for simulation of all valid tests together with the visco-hyperelastic model are shown in Table 4. Curve fits of reduced order model Eq. (19) were done. For low ramp rise times $t_{ramp}$ the response between $t>> t_{ramp}$ and $t_{max}$ showed a nearly constant error, that decays with ramp rise time and is proportional to actuator displacement. This is described with the term $H(x,p_H)$ in reduced order model Eq. (20). The largest time constants identified (Table 3) are in the same order of magnitude as the rest time between individual tests (Table 1). Hence, the influence of residual strains due to the previous tests may not be neglected, and the prior load history term $K(t,p_K)$ takes that into account. The extended reduced order model Eq. (20) achieved a correlation of $R^2=0.99$, and without the $H(x,p_H)$ term of $R^2=0.84$, with respect to the measured force. The last corresponds to the simulated response with the visco-hyperelastic finite element model, which has an $R^2=0.88$ with respect to measured force, and $R^2=0.95$ with respect to reduced order model without $H(x,p_H)$ term. That is, parameters in Table 4 are optimised for simulation with the finite element model, and the reduced order model approximates the simulated visco-hyperelastic response well.

The ramp rise time term $H(x,p_H)$ of Eq. (20) is not depicted in the visco-hyperelastic finite element model. It represents a force that is proportional to the actuator displacement and decays exponentially with ramp rise time, or equivalently with inverse ramp velocity. A hypothesis to explain this effect on the micro scale would be to assume *strain rate hardening*. Higher actuator velocity and corresponding higher strain rate would result in a higher stress level. Strain rate effects were observed by other investigators. ‘With increasing strain rate, we observe a significant increase in fibril modulus and a reduction of fibril to tissue strain ratio, revealing that tissue-level stiffening is mainly due to the stiffening of collagen fibrils.’^26^ The underlying microstructural mechanism are subject of ongoing research^26–28^. In addition, for the PDL compression behaviour^29^ and recovery of the tissue in vivo could play a role. Observe in Table 1 that the test sequence was not randomised with respect to ramp rise time and actuator displacement. The properties of the specimen may have altered during the test and it can not be ruled out that the argued effect is due to test sequence. To clarify this objection further tests with randomised test sequence are recommended (this recommendation is generally valid^25^). Nevertheless, the recent publications cited above support the strain rate hardening hypothesis.

The parameters of the hyperelastic constitutive model for compressible materials identified here ($\mu =0.0338$, $\alpha =29.7$, $\nu =0.236$) confirmed previous results of the authors^8^. They are in good agreement with the values reported in literature^14^ ($\mu =0.03$, $\alpha =20.9$, $\nu =0.257$). Namely $\mu$, which is equal to the initial shear modulus *G*, and the Poisson’s ratio $\nu$, coincide well. For the exponent $\alpha$ a larger difference is observed. This controls the progression of the stress-strain curve. In the simulation of Bergomi et al.^14^ a poro-hyperelastic model was used. That is, the transient response is governed by Darcy’s Law and the long-term response is determined by the hyperelastic constitutive model, i.e. their value is related to the long-term response. By contrast, the value identified here is related to the instantaneous response of the visco-hyperelastic model. This explains part of the difference observed in the exponent $\alpha$.^8^

The poro-visco-hyperelastic simulation shows a slight increase of the peak force versus the visco-hyperelastic response (Fig. 7). The response is practically identical shortly after the initial peak. Figure 9 shows the difference between poro-visco-hyperelastic and visco-hyperelastic peak force for tests with actuator displacement of 0.2$\hbox {mm}$. The difference decreases with ramp rise time, or rather increases with actuator velocity. Considering flow velocities shows that flow of pore fluid vanishes after the initial peak force. This observation is also anticipated from dimensionless analysis. This means that poroelastic behaviour gradually ceases as viscoelastic relaxation progresses. Variation of the poroelastic model parameters within physically justified limits does not alter this behaviour significantly^8^. The contribution of the flow of pore fluid to the poro-visco-hyperelastic response does not coincide with the characteristic of the additional term $H(x,p_H)$ of the reduced order model Eq. (20). This leaves the above argued strain rate hardening effect open and no final permeability parameters were concluded.

In sections of the periodontal ligament (Fig. 1) collagen fibre bundles, interstitial areas and blood vessels are observed. Between the fibre bundles and fibrils there may be free interstitial fluid. The simulation showed that the poroelastic flow of pore fluid is expected in the initial phase of the test^6^, before visco-hyperelastic response prevails. The measurement of Bergomi et al.^14^ showed the presence of interstitial fluid flow. Their porosity value adopted here was ‘assessed by measuring the volume of fluid blotted out of the PDL under a compressive force’, which might have contained vascular fluid. This raises the question of whether the contribution of the vascular system should be modelled. For bone tissue, a vascular relaxation time of $1.36{\mathrm{\upmu }}\hbox {s}$ was stated^4^, and said that ‘even under accidental impact loading, the stress rise time never approaches the vascular porosity relaxation time’. Provided this holds true, the contribution of the vascular system to the reaction force is marginal. However, the voids of the vascular system will compress with little effort. Material parameters identified here indicate a high initial compressibility and it is argued that the voids of the vascular system contribute to that. In addition, ‘Opposite to the behavior expected for a poroelastic material, the tissue volume of different collagenous membranes is observed to strongly decrease with tensile loading.’^30^, could add to the initial compressibility.

It is a truism to say that the experimental setup should represent the periodontal ligament behaviour in vivo. Careful consideration of the notion of *characteristic length* from dimensionless analysis, the concept of *representative elementary volume* (REV), and whether the *boundary conditions* are representative may be of merit to assess experimental setups.

A promising route are nanoindentation experiments, which allows to assess variation of micromechanical properties with tissue composition and location. An investigator used a spherical indenter of radius 5 ${\mathrm{\upmu }}\hbox {m}$ for indentation tests of PDL^31^. This may be considered with respect to the REV. The indenter diameter is smaller than the interstitial areas observed in the PDL section. Therefore, measured values on the micro scale may depend on the location and direction of indentation. For nanoindentation of articular cartilage, ‘there was a high spatial variation and a small change ... in location could change the modulus values up to as much as 10-20 fold.’^32^ Further, ‘The poroelastic relaxation time is quadratic in the radius of the contact.’^33^, i.e. relaxation time depends on the indenter. An obvious choice for the characteristic length of nanoindentation experiments is the diameter of the indenter and parameters may relate to this length scale. How parameters of nanoindentation experiments influence the measured properties and how these are related to the effective (average) properties at the macro scale remains to be clarified.

Several investigators use transverse sections of the PDL (thickness in the order of magnitude of 0.2$\hbox {mm}$) and apply load perpendicular to the plane of section. Hence, the specimen is predominantly loaded in shear, and it should be noted that pure shear deformation does not involve dilation. Examples are^10^, who fitted a quasi-linear viscoelastic model to test data, and^34^ who proposed a ‘creep and relaxation hyper-viscoelastic’ constitutive model. The transverse section leaves two free cut surfaces with ambient pressure boundary condition. This permits free draining of the specimen and alters the dominant direction of interstitial fluid flow towards the free surface. In vivo interstitial fluid flow is expected towards the alveolar bone^14^, that is, roughly in the direction of Sharpey’s fibres. This notion is supported by argument that for ligaments, ‘The permeability in fibre direction is about 2.5 times greater than perpendicular to the fibre direction’^4^. In brief, the influence of the the free boundary, and the position and orientation of the sections with respect to the micro structure, should be kept in mind.

An investigator did harmonic tension-compression tests at 0.1, 0.5 and 1$\hbox {Hz}$ on hydrated bovine periodontal ligament, with cylindrical specimen of diameter 5.8$\hbox {mm}$^14^. The specimen is well one order of magnitude larger than the micro structure observed, i.e. it fulfils the condition for REV. Flow through the free cut surface was observed. However, given the diameter of the specimen, the flow through the alveolar bone is deemed dominant. Flow through the alveolar bone was also observed in the poro-visco-hyperelastic simulation here and it is argued that this is representative for the in vivo condition. The time from zero to the first maximum displacement was 0.025, 0.125 and 0.25$\hbox {s}$. This is smaller than the shortest ramp time used here (min. 5.0$\hbox {s}$). For fast loading, from Fig. 9 a increased contribution of pore fluid flow to the reaction force is expected. Atomic force microscopy based nanoscale rheology investigations of murine PDL observed, ‘increased poroelastic behavior ... at high frequencies’^35^. In summary, for fast loading or high frequency excitation, the contribution of poroelastic flow is gaining importance.

The focus of this work is on the macroscopic response of the PDL to an external load in the initial phase of orthodontic tooth movement. The three parameters of the hyperelastic constitutive model identified are suitable to reflect the averaged, macroscopic response of the PDL. Referring to basic text books^1^, it is well understood that the microstructure of the PDL, and thus local properties at micro scale, vary with anatomic area. Given the complex geometry of the teeth and varying mastication loads it is fair to assume that in vivo the PDL will adapt locally to the predominant load environment. In a paper the ‘significant differences between the collar and furcation regions, with the collar acting as a stabilizing ligamentous structure and the furcation acting as ... a compressive cushion for vertical forces’^35^ was pointed out. Considering alveolar crest and gingival (dentoperiosteal, dentogingival, alveologingival and circular) fibre bundles, together with the above mentioned inhomogeneity of the permeability with respect to fibre direction, it is argued that in vivo the PDL is sealed at the alveolar crest due to this dense network of fibre bundles. A parameter study has shown that alveolar bone permeability has a significant influence on the flow of pore fluid in the periodontium^8^. It is argued that in vivo alveolar bone perforation may adapt locally to optimise for the predominant load situation.

## Conclusion

The initial response of the periodontal ligament to an external load can be simulated with a poro-visco-hyperelastic model. Parameters identified for the hyperelastic constitutive model are in good agreement with published values. They indicate a high initial compressibility of the PDL, which may be attributed to the compressibility of the vascular system within the PDL. The dimensionless analysis indicates that poroelastic behaviour will gradually cease when viscoelastic relaxation progresses. This was as well observed in the simulation and confirmed by variation of the poroelastic model parameters within physically justified limits. Alveolar bone permeability has a significant influence on the flow of pore fluid in the periodontium due to poroelasticity. It is argued that in vivo alveolar bone perforation may adapt locally to optimise for the predominant load situation. A strain rate hardening effect was observed, which is not covered by the simulation, and may be subject of further investigations. Further test with different specimen, loading conditions and randomised test sequence are recommended to confirm the findings. If initial response to fast loading is not of interest a visco-hyperelastic model may suffice.

## Supplementary Information

Supplementary Information.

## Data Availability

Data supporting the results of this work are available on reasonable request from the corresponding author.

## Appendix

### Derivation of the uniaxial stress-stretch relation Eq. (16)

For an introduction to solid mechanics refer to textbooks^5,37^. Assume an *isothermal process* and a *homogeneous, isotropic material*. With that, $J_{th}=1$, $J_{el}=J$, and $\hat{\lambda }_i = \lambda _i$. The strain energy density function, Eq. (7), simplifies to

21 $$\begin{aligned} U\left( \lambda ,J \right) = \sum _{i=1}^N \frac{2\mu _i}{\alpha _i^2} \left[ \left( \lambda _1^{\alpha _i} + \lambda _2^{\alpha _i} + \lambda _3^{\alpha _i} -3 \right) + \frac{1}{\beta _i} \left( J^{- \alpha _i \beta _i} - 1 \right) \right] . \end{aligned}$$

The principal Cauchy stresses $\sigma _k$ are

22 $$\begin{aligned} \sigma _k = J^{-1} \lambda _k \frac{\partial U}{\partial \lambda _k} , \qquad \text {with} \qquad k \in \{1,2,3\} \,. \end{aligned}$$

This requires an estimate of ${\partial U} / {\partial \lambda _k}$. With the basic relation ${\partial J} / {\partial \lambda _k} = J \lambda _k^{-1}$ the derivative follows readily

23 $$\begin{aligned} \frac{\partial U}{\partial \lambda _k} = \sum _{i=1}^N \frac{2\mu _i}{\alpha _i} [ \lambda _k^{\alpha _i-1} - J^{- \alpha _i \beta _i} \lambda _k^{-1} ] , \qquad \text {with} \qquad k \in \{1,2,3\} \,. \end{aligned}$$

To describe the stress tensor $\sigma _{ij}$ index notation and an orthonormal coordinate system, with unit vectors $e_i, \, i\in \{1,2,3\}$ in the 3 coordinate directions, are used. Without loss of generality, assume the *uniaxial state of stress* is in the direction of $e_1$, then $\sigma _{ij}\ne 0$ for $i=j=1$ and $\sigma _{ij}=0$ otherwise. The principal stresses are $\sigma _1=\sigma _{11}$ and $\sigma _2=\sigma _3=0$, and with Eq. (22), this implies

$$\begin{aligned} 0 = [ \lambda _2^{\alpha _i-1} - J^{- \alpha _i \beta _i} \lambda _2^{-1} ] \,. \end{aligned}$$

The principal stretches are $\lambda _1$ and $\lambda _2=\lambda _3$. The Jacobian *J* is equal to the determinant of the deformation gradient *F*. Then $J=det(F)=\lambda _1 \lambda _2 \lambda _3 = \lambda _1 \lambda _2^2$ is substituted in the above equation and we find,

24 $$\begin{aligned} \lambda _2 = \lambda _1^{-\frac{\beta _i}{1+2 \beta _i}} \,, \end{aligned}$$

and the Jacobian becomes

25 $$\begin{aligned} J = \lambda _1 \lambda _2^2 = \lambda _1^{\frac{1}{1+2 \beta _i}} \,. \end{aligned}$$

Consider a *uniaxial tension* test. Assume that the specimen is aligned with, and the load is applied in the direction of $e_1$. Nominal (engineering) stress $\sigma _{nom}$ is defined as force *F* per initial, undeformed cross section area $A_0 = l_{20} l_{30}$ (where $l_{20}$ and $l_{30}$ are the initial lengths in $e_2$ and $e_3$ direction). With current, deformed area $A = l_2 l_3$, and $\lambda _2 = l_2 / l_{20}$, $\lambda _3 = l_3 / l_{30}$, the nominal stress is

$$\begin{aligned} \sigma _{nom} = \frac{F}{A_0} = \frac{F}{A} \frac{A}{A_0} = \sigma _{11} \lambda _2 \lambda _3 = \sigma _1 \lambda _2^2 . \end{aligned}$$

The force per current, deformed area is the Cauchy (true) stress, here the first principal Cauchy stress. With Eq. 22 we find

$$\begin{aligned} \sigma _{nom} = \lambda _2^2 \sigma _1 = \lambda _2^2 J^{-1} \lambda _1 \frac{\partial U}{\partial \lambda _1} , \end{aligned}$$

and, with Eqs. (23) and (25),

26 $$\begin{aligned} \sigma _{nom} =\frac{\partial U}{\partial \lambda _1} = \frac{1}{\lambda _1} \sum _{i=1}^N \frac{2\mu _i}{\alpha _i} [ \lambda _1^{\alpha _i} - J^{- \alpha _i \beta _i} ] \,. \end{aligned}$$

Finally, after some algebra, with $N=1$, Eq. (25), and for the sake of simplicity drop the subscript one, we arrive at Eq. (16), the uniaxial stress-stretch relation

$$\begin{aligned} \sigma _{nom} = \frac{2\mu }{\alpha } \left( 1 - \lambda ^{-\alpha \frac{1+3\beta }{1+2\beta }} \right) \lambda ^{\alpha -1}. \end{aligned}$$
