# Supplementary material for: Towards a reduced order model of the periodontal ligament
Source: Sci Rep. 2025 Feb 17;15:5779. doi: 10.1038/s41598-025-88767-x (PMC11833108; doi:10.1038/s41598-025-88767-x)
Supplement: Supplementary file 1 — Supplementary Information. [file 41598_2025_88767_MOESM1_ESM.pdf]

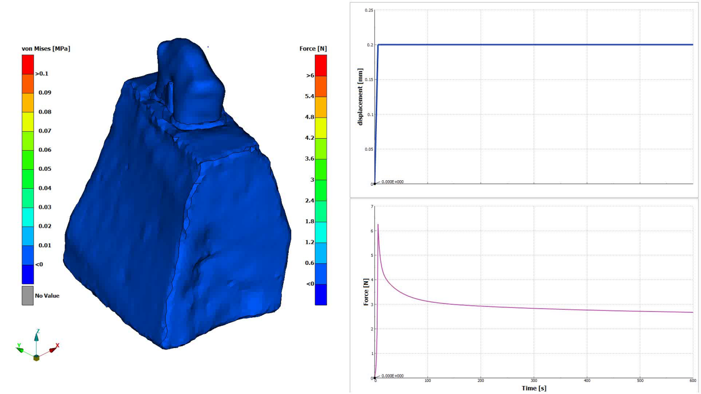

Figure 1: left: displacement, von Mises stress and actuator force; right: actuator displacement and force over time (time-lapse accelerated to the right)

Animation of a Poro-Visco-Hyperelastic Simulation

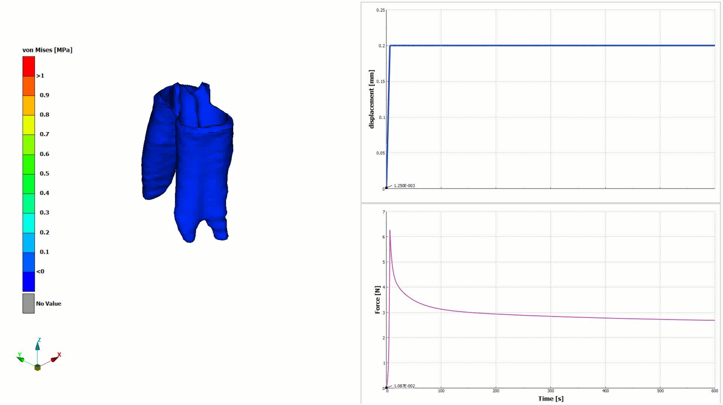

Figure 2: left: von Mises stress; right: actuator displacement and force over time (time-lapse accelerated to the right)

Animation of a Poro-Visco-Hyperelastic Simulation

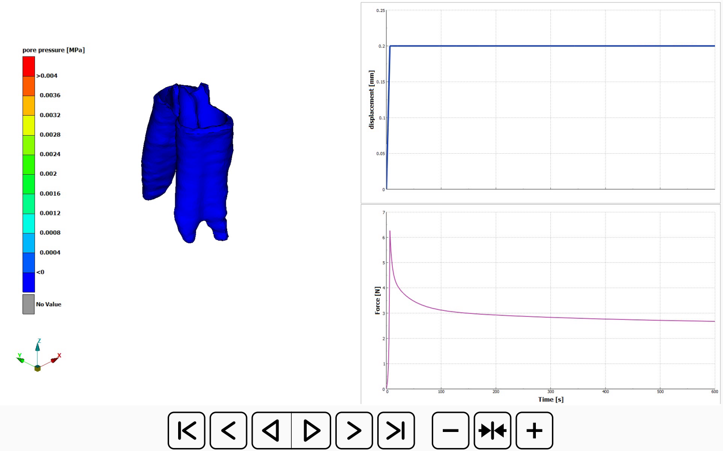

Figure 3: left: pore pressure; right: actuator displacement and force over time (time-lapse accelerated to the right)

Animation of a Poro-Visco-Hyperelastic Simulation

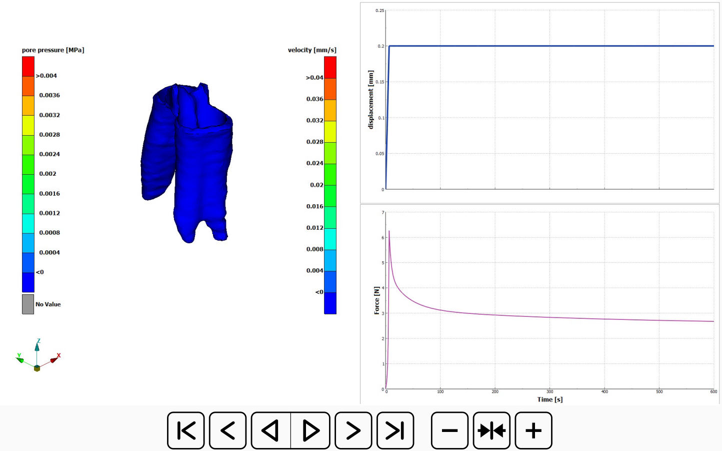

Figure 4: left: pore pressure and pore fluid velocity; right: actuator displacement and force over time (time-lapse accelerated to the right)
